# Recommendations for the Diagnosis and Treatment of Genitourinary Syndrome of Menopause from the Italian Society of Menopause (SIM) and Italian Society of Gynecology of the Third Age (SIGiTE)

**DOI:** 10.3390/healthcare14193205

**Published:** 2026-09-28

**Authors:** Filippo Murina, Costantino Di Carlo, Angelo Cagnacci, Marco Gambacciani, Anna Capozzi, Francesca Nocera, Stefano Lello, Executive Committee of the Italian Society of Menopause (SIM), Italian Society of Gynecology of the Third Age (SIGiTE)

**Affiliations:** 1 Lower Genital Tract Disease Unit, V. Buzzi Hospital, University of the Study of Milan, 20154 Milan, Italy; 2 Department of Public Health, University of Naples Federico II, 80131 Naples, Italy; cdicarlo@unina.it; 3 Teaching Unit of Obstetrics and Gynecology, DINOGMI San Martino Hospital of Genova, 16132 Genova, Italy; angelo.cagnacci@unige.it; 4 Menopause and Osteoporosis Unit, San Rossore Clinical Center, 56122 Pisa, Italy; 5 IRCCS A. Gemelli University Polyclinic Foundation, 00168 Rome, Italystefano.lello@guest.policlinicogemelli.it (S.L.); 6 Independent Researcher, 9521 Catania, Italy

**Keywords:** genitourinary syndrome of menopause, vulvovaginal atrophy, menopause, local estrogen therapy, sexual health, dyspareunia

## Abstract

Genitourinary syndrome of menopause (GSM) is a chronic condition associated with estrogen and androgen deficiency that affects the vulva, vagina, urethra, and bladder, significantly impairing quality of life, sexual wellbeing, and urinary function. Despite its high prevalence, GSM remains frequently underdiagnosed and undertreated. These recommendations, developed by the Italian Society of Menopause (SIM) and the Italian Society of Gynecology of the Third Age (SIGiTE), provide updated evidence-based guidance for the diagnosis and management of GSM. The recommendations were formulated through a comprehensive literature review and expert consensus process, with evidence graded according to the Oxford Centre for Evidence-Based Medicine criteria. Diagnosis should rely on both patient-reported bothersome symptoms and objective clinical signs identified during physical examination. Assessment tools such as the Vaginal Health Index and Vestibular Trophic Health Score may assist in evaluating disease severity and monitoring treatment response. Management should be individualized according to symptom severity, patient preferences, contraindications, and treatment goals. Vaginal lubricants and moisturizers represent the preferred first-line option for women with mild symptoms, whereas local low-dose estrogen therapy remains the treatment of choice for women with moderate to severe GSM. Vaginal DHEA, ospemifene, and pelvic floor physical therapy may be considered in selected clinical settings. Emerging therapies, including energy-based devices, injectable treatments, and vaginal oxygen with hyaluronic acid, may represent additional options in selected patients, although further evidence is needed. Special consideration is required for breast cancer survivors, for whom nonhormonal therapies remain first-line, while low-dose vaginal hormonal therapies may be considered after multidisciplinary evaluation.

## 1. Introduction

The genitourinary syndrome of menopause (GSM) is identified as a group of symptoms of the lower genitourinary tract attributed to the reduction of sex hormone levels that occurs with the cessation of ovarian function. The definition of GSM was first introduced in 2013, replacing previously used terms such as vulvovaginal atrophy, atrophic vaginitis, or urogenital atrophy [1]. The introduction stemmed from the need to overcome a reductionist “vaginocentric” view of these menopausal issues in favor of a new “anatomical and functional” perspective involving the entire lower genitourinary tract. However, it is frequently neglected, underdiagnosed, and inadequately treated, despite the existence of safe and effective therapies, resulting in approximately 50% of women with GSM never having utilized any treatment for their symptoms [2,3]. If left untreated, GSM symptoms frequently endure and can significantly detriment an individual’s health, relationships, sexual intimacy, and overall quality of life. The quality of the evidence was rated using the criteria described in the levels of evidence. This document represents an evidence-based expert recommendation developed by the Italian Society of Menopause (SIM) and the Italian Society of Gynecology of the Third Age (SIGiTE). It should not be considered a formal clinical practice guideline developed according to the GRADE methodology but rather a consensus-based recommendation integrating the best available evidence with expert clinical experience.

## 2. Methods

### 2.1. Scope and Objective

The present document was developed by the Italian Society of Menopause (SIM) and the Italian Society of Gynecology of the Third Age (SIGiTE) with the aim of providing evidence-based recommendations for the diagnosis and management of genitourinary syndrome of menopause (GSM). These recommendations represent an update of the previous national recommendations published in 2019 and integrate current scientific evidence with expert clinical opinion. The document should be considered an evidence-based expert consensus rather than a formal clinical practice guideline developed according to the GRADE methodology.

### 2.2. Literature Search Strategy

A structured literature search was performed to identify the most relevant evidence regarding the epidemiology, pathophysiology, diagnosis, and treatment of GSM. Electronic databases, including PubMed/MEDLINE, Scopus, Embase, and the Cochrane Library, were searched for studies published between January 2019 and March 2026, corresponding to the period following the publication of the previous last SIM/SIGiTE recommendations [4]. The search strategy combined Medical Subject Headings (MeSH) and free-text terms related to GSM, including genitourinary syndrome of menopause, vulvovaginal atrophy, vaginal estrogen, estriol, estradiol, vaginal dehydroepiandrosterone, DHEA, ospemifene, lubricants, moisturizers, pelvic floor physical therapy, laser therapy, energy-based devices, urinary symptoms, breast cancer survivors, and sexual dysfunction. In addition, the reference lists of relevant articles and international guidelines were manually screened to identify further pertinent publications.

### 2.3. Evidence Selection

Priority was given to systematic reviews, meta-analyses, randomized controlled trials, prospective observational studies, and international clinical practice guidelines. When high-quality evidence was unavailable, well-conducted observational studies and expert consensus documents were considered. Case reports, conference abstracts without full publication, duplicate publications, and studies not directly addressing GSM diagnosis or management were excluded. Evidence was independently reviewed by members of the writing committee, and only studies considered clinically relevant and methodologically sound were included in the development of the recommendations.

### 2.4. Development of Recommendations and Grading of Evidence

Following the literature review, the members of the SIM and SIGiTE writing committee critically appraised the available evidence and formulated draft recommendations. Each recommendation was discussed during structured consensus meetings until agreement was achieved among the panel members. Recommendations were subsequently reviewed and approved by the Executive Committees of both scientific societies. The strength of the evidence supporting each recommendation was graded according to the Oxford Centre for Evidence-Based Medicine (OCEBM) Levels of Evidence (Table 1). The wording of each recommendation reflects both the quality of the available evidence and the degree of expert consensus. Whenever robust evidence was lacking, recommendations were based on expert clinical opinion and explicitly identified as such.

## 3. Epidemiology of GSM

Many studies have attempted to define the prevalence of GSM using various approaches; however, all are hindered by methodological flaws that prevent a clear understanding of its actual prevalence. Epidemiological studies conducted by hospital outpatient services across Italy indicate that the prevalence of vulvovaginal atrophy in postmenopausal women is between 79% and 81% [5,6,7,8]. Approximately 65% of women experience this condition one year after menopause, with the prevalence rising to 85% five years later. Additionally, about 64% of women with vaginal atrophy also exhibit signs of vulvar atrophy. Vaginal atrophy is also observed in 20% to 55% of younger women aged 40 to 55 years [5,6,7]. The most common symptom of GSM is vaginal dryness, while pain during intercourse ranks as the second most frequent symptom, particularly among younger women, with its prevalence significantly decreasing within 5 to 7 years post-menopause. Women often find vaginal dryness to be the most bothersome symptom, which affects 3% to 10% of postmenopausal women and 25% of perimenopausal women who have not received a definitive diagnosis of GSM [5,6,7]. This underscores the importance of early identification and treatment of GSM signs and symptoms, even during the transitional phase of menopause. Furthermore, the AGATA study [5] reveals that around 60% of postmenopausal women lack a definitive diagnosis of GSM, primarily attributable to inadequate communication with gynecologists. Women frequently avoid addressing their symptoms, perceiving them as a normal aspect of aging, deeming them insignificant for therapy, feeling humiliated, sensing a lack of attention from physicians, or believing that appropriate medicines are unavailable. Moreover, numerous clinicians neglect to ask about GSM symptoms, and those who do frequently minimize the significance of patients’ reports.

## 4. Clinical Manifestations of GSM

The diagnosis of GSM according to NAMS (North American Menopause Society) requires the presence of signs detectable by the clinician during a physical examination and symptoms reported as bothersome by the patient. The most frequently reported symptoms encompass vulvar irritation, dryness, burning, dysuria, dyspareunia, and vaginal discharge. Symptoms are often classified into vaginal/vulvar, sexual, and urinary categories [1] (Table 2). The listed symptoms may manifest either alone or together, and they may not consistently correspond with the existence or intensity of symptoms; it is crucial to comprehend how each impacts the woman’s quality of life. External genital features may encompass delicate and pallid vulvar integument. Sparse pubic hair and minor labial fusion or reduction in adipose tissue may be observed [3]. The clitoral hood may shrink, revealing the glans, which can result in heightened discomfort during sexual stimulation [8]. A urethral caruncle, characterized by proliferative, bright red tissue near the urethral orifice, may be observed. Posterior fissuring at the introitus may be noted or arise as a consequence of the examination. Extended atrophy may cause introital constriction and fragility, perhaps resulting in discomfort and lacerations during sexual intercourse or the insertion of a speculum during pelvic examination. The vagina may display diminished or missing rugae, little secretions, or potentially yellow, viscous discharge [8].


**Key points**—Recommendation on clinical manifestations.The diagnosis of GSM should be based on both patient-reported bothersome symptoms and objective clinical signs observed during physical examination. Clinicians should assess symptoms across vaginal/vulvar, urinary, and sexual domains, recognizing that signs and symptoms may not always correlate *(Expert Opinion)*.


## 5. Physiopathology of GSM

The key in the pathophysiological mechanism of GSM is hypoestrogenism, which occurs as a result of the cessation of ovarian cycling. Vaginal hypoestrogenism leads to an alteration in the maturation process of the multilayered epithelium, particularly affecting the superficial cells. The reduction in epithelial thickness causes an increased susceptibility to trauma, even of minor severity (such as a cotton swab or a PAP test), resulting in the formation of petechiae, ulcers, and bleeding [9]. Hypoestrogenism also leads to a reduction in glycogen production by vaginal epithelial cells, resulting in a modification of the microbiome with a significant decrease in populations of lactobacilli. This leads to a decrease in vaginal lactic acid production, resulting in an increase in pH, which often becomes higher than 5. The depletion of lactobacilli also promotes the proliferation of pathogenic bacteria, resulting in vaginitis and urinary tract infections. At the local level, vascularization decreases, causing tissues to appear pale, and the quantity of transudate and cervical mucus also decreases, resulting in a sensation of dryness. The caliber and length of the vaginal canal decrease, and elasticity is lost [10]. Estrogen receptors have also been found in the urethra and bladder trigone; therefore, estrogen deficiency can promote the onset of urinary symptoms such as urgency, dysuria, and recurrent urinary tract infections due to the microstructural alterations and changes in the microbiome that occur [11]. Similar to the vaginal wall, some important histological changes also occur in the urethra, such as reduction in vascularization, neurophysiological impairment and connective tissue modifications, contributing to a reduction in urethral tone and, consequently, an increase in urinary incontinence [12]. It has been observed that not only is hypoestrogenism is responsible for the signs and symptoms of GSM, but androgen deficiency also plays a central role in the pathophysiological mechanism. In the postmenopausal period, not only does estrogen production decrease, but also dehydroepiandrosterone (DHEA) production decreases, which is converted intracellularly into both estrogens and androgens; the latter play an active role in maintaining the microarchitecture of the lower genital tract [13]. The vulvar vestibule appears to be significantly involved in genital sensitivity, contributing to atrophy-related superficial dyspareunia in women with GSM [14]. The non-keratinized stratified squamous epithelium envelops a lamina propria abundant in blood vessels, neurons, collagen, elastin, and mucinous glands [15]. In contrast to the primarily autonomic innervation of the vagina, the vestibule is extensively innervated by both autonomic fibers (originating from the pelvic nerves) and sensory somatic fibers (via branches of the pudendal nerve), facilitating pleasurable tactile and pressure sensations during intercourse, and also contributing to chronic pain and allodynia in pathological conditions like vestibulodynia [14]. The vestibule facilitates mucinous lubrication during intercourse, produced by multiple minor glands and two main glands (Bartholin’s glands), and is situated medially adjacent to the vaginal and urethral openings.


**Key points**—Recommendation on pathophysiology.GSM is primarily driven by hypoestrogenism, leading to epithelial thinning, microbiome disruption, and mucosal fragility. Androgen deficiency also contributes, particularly affecting vestibular structure and sensitivity. These hormonal changes result in vaginal, vulvar, and urinary symptoms. Clinicians should recognize the multifactorial pathophysiology to guide personalized, hormone-sensitive treatments. Addressing both estrogen and androgen decline is essential for comprehensive care *(Expert Opinion)*.


## 6. Diagnosis of GSM

The diagnosis of GSM is primarily clinical, relying on a thorough history and physical examination. Physicians should approach the topic sensitively, ensuring patients feel comfortable and at ease during discussions. It is advisable to conduct the initial conversation with the patient dressed, followed by a comprehensive history-taking session encompassing a detailed description of symptoms, sexual history—including arousal, desire, and orgasm—and any attempted self-treatments. Additionally, a gynecologic history is crucial. The physical examination should include an external visual evaluation, musculoskeletal and sensory evaluations, and a bimanual examination if tolerated by the patient [15]. However, a blood test cannot confirm or disprove a GSM diagnosis, as no definitive level of sex steroids has been established. The AGATA study used three parameters to make the diagnosis of GSM, mainly related to the vaginal aspect: a vaginal pH > 5; one objective aspect of the vagina, either dryness, thinning, pallor, fragility or petechiae; and the presence of the subjective sensation of dryness [6]. The estimated prevalence was 79%, similar to another study in the Italian population using a more troublesome evaluation of GSM via the vaginal health index (VHI) [16]. The simple sensation of vaginal dryness identified 97%, and the objective sign of a dry mucosa identified 99% of the GSM cases [6]. VHI [17] and Vestibular Trophic Health (VeTH) [18] are two tools that can help measure how bad the condition is and how well the patient is responding to treatment. Specifically, the VHI is mainly designed to evaluate vaginal trophism and assesses five domains: vaginal elasticity, secretions, pH, epithelial mucous membrane, and hydration, providing a standardized measure on a numerical scale to track changes over time [17]. The total score ranges from 5 to 25, with lower scores indicating greater GSM severity. On the other hand, VeTH has been designed mainly to evaluate vestibular trophism, and it assesses five criteria: petechiae, pallor, thinning, dryness, and redness. The vulvar vestibule, being rich in nociceptors, is a primary site of genital tenderness [14]. This sensitivity often manifests as burning, pain, and dyspareunia upon entry, especially in cases of sexual hormone deprivation, with a significant correlation between VeTH scores and the severity of symptoms experienced by patients. Morpho-functional changes are also linked to a drop in circulating steroids, glycogen, and lactic acid. This causes the pH to rise and dysbiosis to occur, which then shows up as a variety of symptoms [19]. These symptoms include decreased vaginal lubrication, vaginal dryness, dyspareunia, loss of libido, sexual dysfunction, infections, burning, itching, bleeding, leucorrhea, urinary incontinence, urinary urgency, dysuria, and vaginitis.


**Key points**—Recommendation on diagnosis.GSM The diagnosis of GSM is clinical and should be guided by a sensitive, structured history and physical examination. While the diagnosis is rather simple and can be based on anamnesis and a simple physical examination, careful assessment of vaginal and vestibular health using validated tools like the Vaginal Health Index (VHI) and Vestibular Trophic Health (VeTH) is useful for evaluating severity and treatment response. Laboratory tests are not diagnostic, and attention should be given to symptom clusters that impact quality of life, including sexual and urinary dysfunction *(Expert Opinion)*.


## 7. Treatment of GSM

The objective of GSM treatment is to reduce symptoms and improve lower urogenital function. Therapy for GSM requires personalized and collaborative decision-making, with treatment beginning based on the effect of symptoms on the woman’s personal and sexual needs, rather than only on physical assessments. This approach ensures that the therapy is not only effective in addressing physical symptoms but also enhances overall quality of life. By prioritizing the individual’s experience, healthcare providers can create a more supportive and responsive treatment plan. A tailored and integrated approach to treatment for GSM emphasizes the importance of customizing therapeutic interventions to meet the unique needs and preferences of each woman. Post-menopause, the vulva and vagina are more susceptible to friction and irritants. It is crucial to educate women about vulvovaginal hygiene, emphasizing the importance of selecting products with mild surfactants and steering clear of those that contain fragrances. This method maintains the fragile equilibrium between skin cleanliness and the integrity of the stratum corneum barrier. Compromise of this barrier may result in heightened trans-epidermal water loss and a reduction of the natural moisturizing factor. Consistent sexual stimulation can be advantageous, since it enhances vaginal blood circulation and secretions, whether performed with a partner, solo, or via a gadget (e.g., vibrator). These behaviors can improve hormonal and nonhormonal treatment procedures by alleviating further irritation of the delicate vulvovaginal tissues [20].

### 7.1. Lubricants and Moisturizers

The principal therapies for mitigating symptoms of GSM include non-hormonal vaginal lubricants and moisturizers; however, limited clinical studies have been conducted to evaluate their effectiveness [21]. Four small to moderate-sized trials evaluated vaginal moisturizers against placebo over a 12-week period. The formulations and dose schedules of vaginal moisturizers differed. Overall, low-certainty evidence indicates that vaginal moisturizers may have negligible or no effect on dyspareunia compared to a placebo. The moisturizer group demonstrated a more pronounced enhancement in vulvovaginal dryness than the placebo group; however, the statistically significant difference in improvement was observed in only two of the trials. Both lubricants and moisturizers alleviate the friction caused by thin, dry genital tissue due to vulvovaginal GSM. Investigations into personal moisturizers and lubricants indicate that certain water-based solutions are hyperosmolar, potentially causing epithelial cellular toxicity and damage. Near iso-osmolar and silicone-based lubricants do not demonstrate this effect. The World Health Organization advises an osmolarity of under 1200 mOsm/kg [22]. No published reports exist on the irritation potential of over-the-counter vaginal lubricants and moisturizers. The primary difference between vaginal moisturizers and lubricants is their intended use. Moisturizers are designed to hydrate and restore vaginal tissues over a prolonged duration, whereas lubricants are used as needed, administered directly to the genital area, generally prior to or during sexual intercourse. Vaginal hyaluronic acid (HA) functions mainly through its hydrating qualities, epithelium healing, mucoadhesion, and anti-inflammatory processes. Although numerous studies suggest that hyaluronic acid may be advantageous for the therapy of GSM, these investigations are constrained by their modest sample sizes and methodological rigor. In four randomized controlled trials, hyaluronic acid showed a similar improvement in dryness and dyspareunia when compared to placebo or vaginal estrogen therapy [23]. At present, there is no evidence suggesting that products containing hyaluronic acid offer superior benefits compared to lubricants or moisturizers devoid of hyaluronic acid. For women with minor vaginal dryness or dyspareunia, vaginal lubricants and moisturizers may serve as an effective first-line treatment. Although these products may provide instant relief and improve comfort during sexual activity, they should be considered components of a more comprehensive supportive treatment strategy. Additional steps should be contemplated to comprehensively address these symptoms, as exclusive dependence on lubricants and moisturizers may prove inadequate for all individuals. Investigating additional supporting alternatives may facilitate a more thorough alleviation of symptoms.


**Key points**—Recommendation on lubricants and moisturizers.Vaginal lubricants and moisturizers can be recommended as a first-line, non-hormonal treatment for women with mild GSM symptoms, particularly for relieving vaginal dryness and dyspareunia. Evidence from small to moderate RCTs suggests modest benefit, though with low certainty. Products should be selected carefully, prioritizing iso-osmolar formulations to minimize epithelial damage. These therapies are best used as part of a broader, individualized management plan, not as stand-alone solutions *(Strength of Recommendation: Moderate)*.


### 7.2. Vaginal Estrogen Therapies

For individuals presenting symptoms limited to GSM, local estrogen therapy is the optimal choice, as vaginal administration provides sufficient estrogen to reduce symptoms with minimal systemic absorption, rendering it more favorable than systemic therapy [1,8]. Furthermore, around 40% of women undergoing hormonal systemic therapy may continue to experience symptoms of GSM and could benefit from the addition of vaginal estrogen [8]. However, the labeling for vaginal estrogen products now features the same boxed warnings as those for systemic therapy. Clinical trials evaluating the efficacy of vaginal estrogen products showed their superiority compared to placebo in objective measures, including the maturation of the vaginal epithelium, as well as in alleviating symptoms of vaginal dryness and urogenital issues, such as increased urinary urgency and decreased incidence of cystitis [24]. Low-dose vaginal estrogen therapies exhibit considerable efficacy for patients with GSM, resulting in a 60% to 80% decrease in symptom severity, as demonstrated by various trials [20]. A variety of prescription vaginal estrogen products are available in Italy, as detailed in Table 3. All products, except vaginal rings, necessitate an initial loading dosage involving daily administration for nearly three weeks, followed by maintenance dosing of two to three times weekly, based on symptom management [1,8]. Vaginal estrogen should be administered at the minimal effective dosage for the period of documented advantages and may be utilized indefinitely. All low-dose vaginal estrogen formulations exhibit little absorption and do not require the prescription of a progestogen for endometrial protection [1]. The safety of low-dose vaginal estrogen is corroborated by multiple randomized controlled trials and long-term observational studies, indicating no elevated risk of cancer (breast, endometrial, ovarian, or colorectal) [25], and comprehensive observational analyses found no correlation between vaginal estrogen use and an elevated risk of venous thromboembolism [26]. The selection of formulation—creams, gels, tablets, or rings—must be tailored to the patient’s comfort and convenience, facilitating effective symptom management while minimizing risk. Absorption of local estrogen varies by the active ingredient (potency: conjugated equine estrogens > estradiol > estrone > estriol). It also varies according to the amount of active ingredient applied (e.g., creams applied to a greater surface area in the vagina are more readily absorbed than vaginal tablets or rings) and where the estrogen is applied, whether only in the vagina or even on the vulvar vestibule [27]. No significant difference in efficacy has been observed among the various intravaginal estrogenic preparations for managing GSM symptoms [28]. However, estriol should be preferred because although it is a weaker estrogen compared to estradiol, it has proved to be equally effective in alleviating both subjective and objective symptoms of GSM [28]. It binds less strongly to estrogen receptors, and its effects are more localized when administered vaginally; it acts locally with minimal systemic absorption and a better safety profile. In the treatment of GSM, local therapies have traditionally focused on the vaginal area. However, recent research highlights the crucial role of the vestibule [29,30]. Symptoms such as dryness and introital dyspareunia, which are characteristic of this syndrome, are increasingly recognized as originating from alterations in the vestibular area. Consequently, it is imperative to provide therapy at this specific location as well. In summary, local estrogen therapy effectively addresses GSM symptoms, offering various formulations to accommodate individual preferences. Although systemic absorption is minimal, it is important to administer the lowest effective dose and to monitor for potential adverse effects.


**Key points**—Recommendation on vaginal estrogen.Local estrogen therapy is the treatment of choice for women with symptomatic GSM, offering high efficacy with minimal systemic absorption. All low-dose formulations (creams, tablets, gels, rings) are equally effective; estriol may be preferred due to its lower systemic activity. Application should include the vestibule when symptoms such as introital dyspareunia are present. Long-term use is safe and does not require *progestogen (Strength of Recommendation: Strong)*.


### 7.3. Androgens

#### 7.3.1. Vaginal Dehydroepiandrosterone (DHEA)

Local use of DHEA, also sometimes referred to as prasterone, has been shown to reduce vaginal pH and improve the vaginal maturation index, dryness, dyspareunia, and sexual function compared to placebo [31]. Some trials have shown that intravaginal prasterone is equally effective as intravaginal estrogens for treating vulvovaginal atrophy and dyspareunia [32]. Two prospective clinical trials indicated a decrease in stress urinary incontinence (SUI) and urgency episodes, as measured by the 3-day bladder voiding diary, along with an increase in bladder compliance after 12 weeks of treatment with DHEA [31]. The most common intravaginal dosage for DHEA is 6.5 mg daily for 8–12 weeks. The intracrinology mechanism clarifies the localized effects of DHEA, along with findings from multiple studies indicating no significant elevation in blood estrogen and testosterone levels following DHEA administration for up to 12 months, which suggests an almost complete absence of side effects [33]. The mechanism of action of vaginal DHEA is likely associated with its intracrine activity and its role as a prohormone for androgens and estrogens. DHEA is metabolized into androstenedione and testosterone, which can be aromatized in the vaginal mucosal cells to estrone and estradiol [33]. Consequently, it may exhibit agonistic activity at the estrogen and androgen receptors in the vagina [34]. Vaginal discharge was the most frequently reported adverse event, occurring in approximately 6% of women enrolled in various trials. In a large study involving women treated with DHEA for 52 weeks, endometrial sampling revealed an inactive or atrophic endometrium in all participants [35].

#### 7.3.2. Testosterone

The use of topical testosterone in the treatment of genitourinary syndrome of menopause is off-label [36]. The rationale for the application of an androgenic product in the treatment of this syndrome is the presence of a significant concentration of androgen receptors at the vaginal, vestibular, and vulvar levels [37]. Local testosterone, in fact, would act by increasing vascularization, the density of nerve fibers, mucous secretions, and collagen synthesis. The studies conducted have shown clinical improvement in signs of vulvovaginal atrophy, improvement in dryness, and likely improvement in sexual function [37]. Clinical experiences indicate a positive synergistic effect of testosterone combined with topical estrogens on trophism when applied to the vestibular area, which has a high concentration of estrogenic and androgenic receptors. Although bioidentical and derived from hormones, these galenic formulations lack comprehensive efficacy and safety assessments through randomized, placebo-controlled trials, resulting in ambiguities concerning their purity, potency, and safety. Thus, in the absence of official authorization and packaging details, the prescribing physician is accountable for adequately advising the patient of potential side effects, evaluating indications and contraindications, and instructing on the reporting of adverse events [38].


**Key points**—Recommendation on vulvovaginal androgens.Intravaginal DHEA is an effective and safe treatment for GSM, with minimal systemic absorption and no endometrial stimulation. The recommended dose is 6.5 mg daily for 8–12 weeks (*Strength of Recommendation*: *Moderate*). Topical testosterone, though off-label, shows promise due to its action on androgen receptors in vulvovaginal tissues, particularly in the vestibule, and may enhance outcomes when combined with estrogens. However, further studies are needed to confirm its safety and efficacy *(Strength of Recommendation: Expert opinion)*.


### 7.4. Ospemifene

Ospemifene is the only Selective Estrogen Receptor Modulator (SERM) approved by the FDA for the management of moderate to severe dyspareunia and/or vaginal dryness resulting from genitourinary syndrome of menopause [1]. In contrast to other SERMs, such as tamoxifen and raloxifene, ospemifene exhibits a specific affinity for estrogen receptors in the squamous epithelium of the vulvovaginal region [39]. The effectiveness of ospemifene at a dosage of 60 mg/day for alleviating GSM symptoms was evidenced in comparison to placebo at the 12-month follow-up [40]. Since food intake significantly enhances the oral bioavailability of ospemifene, the drug should be taken with meals to ensure adequate absorption [40]. The outcome was corroborated by evidence, including elevated vaginal pH, proportions of superficial and parabasal cells in VHI, sexual function, vaginal histology and morphology, advantages noted in the vulva, vestibule, urethral meatus, and vagina, along with enhancements in the vaginal microbiome and metabolic profile [40,41,42,43]. The effectiveness of ospemifene seems to be similar to that of local estrogen therapy for addressing the genitourinary syndrome of menopause. In addition to its effects on vaginal trophism and dyspareunia, ospemifene has demonstrated benefits on quality of life and sexual function in postmenopausal women with GSM, including improvements in FSFI scores and patient-reported sexual wellbeing [44]. Emerging evidence also suggests a potentially favorable safety profile in women with a history of breast cancer. In Europe, ospemifene may be considered in selected breast cancer survivors after the completion of adjuvant therapy. Baseline data from the PEONY study highlighted the high burden of vulvovaginal atrophy symptoms and impaired quality of life in women with and without a history of breast cancer. Moreover, six-month results from the PEONY-BC study reported improvements in quality of life, treatment satisfaction, and GSM symptoms in breast cancer survivors treated with ospemifene, along with reassuring short-term safety findings. This conclusion arose from a comparative analysis of ospemifene and historical data on local estrogen therapy, despite the absence of direct comparative research [44]. The benefits of ospemifene therapy for urinary symptoms of GSM were assessed in three studies with small sample sizes, largely indicating enhancement in symptoms related to overactive bladder [43].In terms of side effects, ospemifene exhibits a restricted occurrence of adverse effects (AEs) compared to placebo, with a low dropout rate of 1.6% [43]. Hot flashes are prevalent adverse events, occurring in 8.5% of cases compared to 3.3% for placebo. No instances of endometrial carcinoma, hyperplasia, polyps, or cancer were observed after 52 weeks, and the PASS study indicated no elevated risk of venous thromboembolism in patients with ospemifene relative to other SERMs or the control group [44]. No randomized controlled trial has yet investigated the long-term breast safety of ospemifene; nonetheless, preclinical studies and observational data suggest a neutral effect on breast cancer risk. Ospemifene may beneficially affect bone metabolism in postmenopausal individuals. Two randomized controlled trials (RCTs) demonstrated an anti-resorption effect of ospemifene akin to that of raloxifene [43], although only one observational study supplied data on bone mineral density (BMD) [45]. In conclusion, the existing evidence, demonstrating superior efficacy and safety profiles, endorses the utilization of ospemifene as a first-line therapeutic agent for the management of GSM. As healthcare providers evaluate therapy alternatives for patients exhibiting symptoms of genitourinary syndrome of menopause, ospemifene’s advantageous profile may offer a significant alternative to traditional treatments, while enhancing patient quality of life and satisfaction.


**Key points**—Recommendation on ospemifene.Ospemifene is approved for the treatment of moderate to severe dyspareunia and/or vaginal dryness due to GSM. Administered at 60 mg/day, it has demonstrated comparable efficacy to local estrogen therapy in improving vaginal pH, epithelial maturation, sexual function, and microbiome health. With a favorable safety profile—and a low risk of endometrial or thromboembolic events—it represents a valid first-line, non-vaginal alternative for GSM *(Strength of Recommendation: Moderate)*.


### 7.5. Energy-Based Devices

Three different types of energy devices are presently utilized for vaginal therapy in GSM patients, with multiple manufacturers represented in each category [46]. Two of these modalities utilize laser technology (CO_2_ and Erbium:YAG), while the third employs radiofrequency (RF). The functioning mechanism of these devices relies on the principle that a certain amount of thermal energy applied to the vaginal wall exerts a regenerative influence on local tissues, hence promoting collagen synthesis, angiogenesis, and epithelial hypertrophy [47]. The predominant lasers utilized for GSM are fractional micro-ablative carbon dioxide (CO_2_) and non-ablative erbium-doped yttrium aluminum garnet (Er:YAG). Micro-ablative lasers create a grid of minute perforations in the surface tissue. Fractional lasers divide the laser beam into columns, preserving the intervening tissue to expedite recuperation. Non-ablative lasers elevate the temperature of subcutaneous tissue while preserving the integrity of the epidermis [48]. RF devices use electromagnetic currents to generate heat due to tissue resistance to these currents. Heating tissues to temperatures between 42 and 45 °C induces micro-remodeling through heat shock protein cascades, hence activating fibroblasts and commencing neocollagenesis and elastogenesis [47]. Several studies have examined the application of Erbium:YAG in GSM. All trials are observational, with participant numbers varying between 24 and 205. Research indicated the administration of two or three laser treatments at intervals of 3 to 4 weeks, with a maximum follow-up duration of 24 months. All studies indicated a significant improvement in symptoms of GSM, including dryness and dyspareunia. The studies also documented significant modifications in the VHI, female sexual function index (FSFI), and histological findings [48,49]. Thanks to the non-invasive mechanism of action of Smooth technology, intraurethral Erbium:YAG laser can be applied for the treatment of the urethral component of GSM (namely urgency, frequency) [50].

AEs, characterized as mild and transient (vaginal discharge), were observed in up to 4% of patients receiving Erbium:YAG vaginal laser treatment [51]. Research with follow-up durations of 18 and 24 months indicates a decline in benefits after 12 months, implying that repeated treatments may be necessary [48,49]. The evidence validating the application of fractional ablative CO_2_ laser in the therapy of GSM is substantial, consisting of 41 articles, predominantly observational, with only 8 randomized controlled trials (RCTs) [46,49]. The time between treatments is between 2 and 6 weeks, and the time between follow-ups is between 1 and 24 months. Two systematic reviews of fractional CO_2_ lasers indicated significant improvements in both subjective symptoms and objective signs of GSM across 39 out of 41 studies examined [49,52]. The sham-controlled RCTs have yielded inconsistent findings. Two RCTs involving 88 and 58 participants, respectively, indicated significant differences in symptoms and signs. In contrast, two other studies with 30 and 18 participants showed no difference between the treatment and sham groups, potentially highlighting a placebo effect [49,52]. All RCTs exhibited bias due to limited sample sizes, different laser settings, varying outcomes, and differing analytical criteria. Even so, it should be noted that therapeutic failures essentially depend on the inappropriate conduct of studies, the selection of patients, and the failure to adhere to recommended standards. The duration of treatment has been examined in several studies, which indicate that treatment effects remain significant at 12 and 15 months. However, after this period, the benefits may diminish, necessitating additional treatment. Only one trial, involving 84 women with symptomatic GSM, found sustained treatment effects at 24 months [48]. Several studies have compared the effect of vaginal laser and vaginal estrogen on GSM, with no significant difference between the groups in terms of symptom improvement regardless of the type of laser used. Interestingly, a double-blinded RCT with 15 women in each arm compared CO_2_ laser, topical estrogen, and combination treatment, demonstrating a significant improvement in all groups, with the greatest improvement in the combined treatment group [52]. Studies associating the laser with ospemifene and hyaluronic acid have further confirmed the combined synergistic effect [53,54]. A systematic review and meta-analysis evaluated 13 high-quality studies involving 650 patients to assess the efficacy of CO_2_ laser therapy in managing symptoms of SUI in women [55]. The findings showed that CO_2_ laser therapy was effective, as evidenced by significant improvements in various SUI-related measures, including the 1-h pad test, ICIQ-UI SF, PFIQ, UDI-6, and VHIS at follow-up intervals of 3, 6, and 12 months. Additionally, it is important to note that some studies were single-arm investigations that lacked randomization, with only 4 RCTs included, which may have introduced a risk of selection bias in the results. The efficacy of Erbium:YAG laser in the treatment of SUI has also been demonstrated in observational studies with a follow-up of 18–24 months, using the vaginal approach alone or in association with intraurethral treatment [56,57,58,59]. In addition, the clinical effects of erbium Smooth laser treatment have been confirmed in randomized controlled trials with a sham treatment (placebo) [48,60,61,62,63].

A comprehensive review and meta-analysis of four monocentric case series with small patient cohorts (127 participants) undergoing dynamic quadripolar RF of GSM [64]. Two studies reported a significant improvement in patient and medical evaluation assessments of vulvovaginal aesthetic appearance. Notable enhancements were noted in three investigations concerning patient-evaluated sexual satisfaction and discomfort, vaginal laxity, and symptoms related to the genitourinary syndrome of menopause. The meta-analysis revealed a tendency for improvements in aesthetic appearances. These findings, while intriguing, require confirmation through rigorously conducted, large-scale controlled trials. The prevalence of post-procedure side effects reported in the literature shows a high degree of variability; however, no serious complications, including those from CO_2_ and Er:YAG lasers and RF, have been documented [48,49,51]. This makes the prejudice-based fears regarding the use of devices unfounded, as long as they are used appropriately with the correct disease indications. The main adverse effects are vaginal discharge, pain during and post-procedure, itching, and atypical bleeding. All adverse outcomes were classified as mild or moderate, and no permanent complications were reported. The existing reviews and meta-analyses indicate that energy-based devices (EBDs) may serve as a therapeutic intervention capable of reducing symptom severity, enhancing the quality of life for postmenopausal women, and restoring the vaginal mucosa to its premenopausal condition. To advance the understanding of EBD treatments, future research should focus on analyzing the variety of EBD therapies, their applications, the integration of evidence-based approaches with alternative treatments, and the exploration of different dosing strategies and schedules. Based on the available literature, the most commonly adopted protocol consists of three treatment sessions performed every 3–4 weeks for Er:YAG laser or every 2–6 weeks for fractional CO_2_ laser. Clinical improvement generally persists for approximately 12 months, after which maintenance treatment may be considered according to symptom recurrence and patient response. Energy-based devices may be considered in carefully selected women who have failed conventional therapies or are not candidates for hormonal treatment. However, current evidence remains heterogeneous, and further adequately powered sham-controlled randomized trials are needed before routine clinical use can be recommended.


**Key points**—Recommendation on energy-based devices.Energy-based devices (EBDs)—including fractional CO_2_ laser, Er:YAG laser, and radiofrequency—have shown potential in providing symptomatic relief in GSM, particularly for vaginal dryness, dyspareunia, and mild urinary symptoms. Although current evidence is still evolving and somewhat limited by methodological constraints and relatively short follow-up, these approaches may represent a reasonable therapeutic option for selected patients, especially those who decline or are not candidates for hormonal therapies. Ongoing research will help better define their long-term safety and effectiveness, and their use should be guided by individualized clinical judgment and patient preferences *(Strength of Recommendation Moderate)*.


### 7.6. Other Treatments

#### 7.6.1. Injection Treatments

Injection treatments have been proposed as novel therapeutic approaches for vulvovaginal atrophy related to GSM. Various types of injection materials are available: hyaluronic acid (HA) fillers (cross-linked, non-crosslinked, thermally stabilized, and combined with calcium hydroxyapatite), lipofilling (including both nanofat and microfat grafting), platelet-rich plasma (PRP) injection, and the combination of PRP injection with lipofilling [65]. HA treatments function by hydrating tissues and promoting fibroblast proliferation. When combined with calcium hydroxyapatite, non-crosslinked HA further enhances the production of collagen and elastin, activates keratinocytes, and aids in reducing inflammation. PRP and micro-fragmented adipose tissue (MFAT) injections promote tissue regeneration by activating multiple growth factors. This procedure enhances fibroblast proliferation and elevates the synthesis of type I collagen, along with matrix metalloproteinases 1 and 2 [66,67]. These actions facilitate tissue proliferation and differentiation, neoangiogenesis, and the production of a new extracellular matrix, thereby improving trophism. The analysis of the studies indicated an improvement following multiple injection treatment strategies that focused on addressing GSM symptoms, sexual function, and vaginal health [65]. The observed improvement occurred 1 to 2 months after treatment, with effectiveness sustained for a minimum of 3 to 6 months. The variability in treatment outcomes and follow-up durations, the absence of direct comparisons among different injection treatments, and the lack of clarity regarding the injection sites (such as the vulvar vestibule, labia minora, and vagina) limited the ability to draw definitive conclusions regarding the effectiveness of injection strategies relative to other treatment options.


**Key points**—Recommendation on injection treatments.Injectables such as hyaluronic acid, PRP, and lipofilling show promise for improving GSM-related vulvovaginal atrophy by promoting tissue hydration and regeneration. Early studies report symptom relief and improved sexual function within 1–2 months, lasting up to 6 months. However, evidence is limited, heterogeneous, and lacks standardization. These therapies remain experimental and should be used cautiously in select patients *(Strength of Recommendation: Expert opinion)*.


#### 7.6.2. Vaginal High Concentration Oxygen with Hyaluronic Acid

The combination of high-concentration topical oxygen therapy with hyaluronic acid represents a promising non-hormonal treatment for vulvovaginal atrophy, especially in women who are not candidates for hormonal therapies [68,69,70,71]. Preliminary clinical studies have reported significant improvements: one prospective study observed a 70% reduction in symptoms such as dryness and burning after six weeks of treatment, while another pilot study using a vaginal natural oxygenation device noted enhanced epithelial trophism and increased sexual comfort in over 80% of participants [68,69,70,71]. Additionally, improvements in sexual function scores and patient satisfaction were consistently reported across studies. Despite these encouraging results, the current evidence is limited to small-scale trials. Larger, well-designed RCTs are essential to validate these findings, assess long-term safety, and define standardized treatment protocols.


**Key points**—Recommendation on vaginal high-concentration oxygen with hyaluronic acid.The combination of vaginal high-concentration oxygen with hyaluronic acid shows promise as a non-hormonal option for GSM, especially in women unable to use hormones. Preliminary studies report symptom improvement and enhanced epithelial health. However, due to small sample sizes and lack of RCTs, further high-quality trials are needed before widespread clinical adoption *(Strength of Recommendation: Moderate)*.


#### 7.6.3. Pelvic Floor Physical Therapy

Hypertonic dysfunction of the pelvic floor musculature is frequently associated with dyspareunia related to GSM. In these instances, pelvic floor physical therapy can be an effective adjunctive treatment. This therapy utilizes both internal and external techniques, including biofeedback, vaginal myofascial release, and stretching, to promote muscle relaxation, normalize resting muscle activity, and improve vaginal elasticity. Additionally, vaginal dilators may be employed to facilitate mechanical distention and encourage progressive relaxation of the vaginal muscles [72]. Although studies and systematic reviews indicate that pelvic floor muscle rehabilitation can significantly reduce dyspareunia, there is a notable absence of controlled studies specifically targeting postmenopausal subjects. Women should be made aware of the advantages of physical treatment, especially if they have high-tone or non-relaxing pelvic floor muscle dysfunction brought on by painful GSM-related sexual activity.


**Key points**—Recommendation for pelvic floor physical therapy in GSM.Pelvic floor physical therapy may be a beneficial adjunct in managing dyspareunia associated with GSM, particularly in cases of hypertonic pelvic floor dysfunction. Techniques such as biofeedback, myofascial release, and vaginal dilators can improve muscle relaxation and vaginal elasticity *(Expert consensus)*.


## 8. GSM in Women with a History of Breast Cancer

Advancements in breast cancer detection and treatment have led to an increase in female survivors, with a reported 5-year survival rate reaching as high as 90%. GSM is frequently seen in women who have had breast cancer, with up to 75% of breast cancer survivors (BCS) reporting at least one genitourinary symptom [73]. The predominant symptom is vulvovaginal dryness, experienced by over 60% of these women, followed by entry dyspareunia, which impacts nearly 40% [73]. Despite its prevalence, many breast cancer survivors’ oncologists do not advise them that breast cancer treatment may result in genitourinary problems. Symptoms related to the lower genitourinary tract may be triggered or intensified by treatments for breast cancer. Therapies such as chemotherapy, tamoxifen, and aromatase inhibitors (AIs) can worsen a hypoestrogenic condition, leading to decreased estrogen levels in genital tissues. This reduction can adversely affect sexual function and significantly diminish quality of life. Currently, there is no established consensus on how to manage GSM in BCS. Although the estrogen levels of women taking AIs are lower than those usually observed after menopause, they are not immune to the effects of exogenous estrogens. Even though tamoxifen users still have some estrogen in their bodies, the medication prevents it from acting on breast tissue. Nonhormonal products, such as vaginal moisturizers and lubricants, serve as an important first-line therapy for vulvar and vaginal dryness, as well as related dyspareunia, in all women, particularly those with hormone-dependent cancers. However, in many cases, these therapies may not sufficiently alleviate the symptoms. In a randomized study involving breast cancer survivors experiencing severe GSM and dyspareunia, the application of 4% aqueous lidocaine as a nonhormonal treatment option prior to penetration resulted in an 88% reduction in dyspareunia, compared to a 33% reduction with placebo [74]. Despite these promising findings, we do not recommend this method due to the potential damage it may cause to atrophic mucosa, as well as the risk of developing sensitivity to local anesthetics. Currently, there is some hesitation to utilize local estrogen therapy in BCS due to its potential adverse effects. Up to 70% of oncologists who manage BCS refrain from prescribing hormone therapies [75]. There is concern about an increased risk of breast cancer recurrence and possible interference with adjuvant treatments, such as tamoxifen or AIs. As a result, the only recommended therapy for these patients involves the use of moisturizers and lubricants. A new meta-analysis investigated the risks of breast cancer recurrence, breast cancer-specific mortality, and overall mortality among BCS utilizing vaginal estrogens for GSM. The results indicate that vaginal estrogen use is safe in this cohort; notably, vaginal estrogens were not associated with a heightened risk of breast cancer recurrence (24,060 patients, OR 0.48) or breast cancer-related mortality (61,695 patients, OR 0.60) [76]. In a large claims-based analysis involving 10,584 patients, researchers found no significant difference in the risk of cancer recurrence within five years between breast cancer survivors who received prescriptions for vaginal estrogen therapy for a diagnosis of GSM and those who did not. This finding held true regardless of whether the survivors had a history of estrogen receptor–positive cancer [77]. AIs do not prevent the effects of exogenous estrogens on breast tissue, in contrast to tamoxifen. Elevated serum estrogen levels have the potential to stimulate estrogen receptors in breast tissue when estrogen and an AI are used together. However, a review of ten studies that evaluated serum estradiol levels revealed that the majority either showed no increase in serum estrogen levels at all or only a brief increase that went away after 12 weeks [78]. The levels of estriol, estradiol, and estrone did not change after a 12-week treatment with 0.005% estriol vaginal gel in postmenopausal women undergoing treatment with nonsteroidal aromatase inhibitors for hormone receptor-positive early breast cancer [79]. Studies evaluating the safety of FDA-approved DHEA in cancer survivors are limited. In one randomized controlled trial involving women who survived breast cancer and experienced GSM, participants who received vaginal DHEA reported significant improvements in sexual health. The authors noted that serum levels of DHEA-S and testosterone were significantly increased in these women. However, estradiol levels remained within the lower half of the postmenopausal range, and there was no increase in serum estradiol levels among women who were on AIs [80]. Clear communication with a patient’s medical oncologist, when feasible, will enhance the shared decision-making process. Currently, there is no evidence suggesting harm from these local treatments, especially low-dose vaginal estrogen. Ospemifene is indicated for women in Europe who have a history of breast cancer and have completed treatment [78]. A total of 185 patients were included in three RCTs that compared CO_2_ laser, radiofrequency, and Er:YAG laser treatments to sham laser, promestriene, and hormone-free vaginal suppositories. These patients reported an overall improvement in dyspareunia with physical energy methods, with effects lasting up to six months and minimal adverse events. However, the improvements did not reach statistical significance between the intervention and control groups. In contrast, data from a retrospective study that included both healthy women and BCS demonstrated statistically significant improvements in dyspareunia and vaginal dryness using the same energy sources [81]. Recent evidence also suggests that the combination of vaginal oxygen therapy and hyaluronic acid may improve lower urinary tract symptoms and vaginal health in breast cancer survivors with GSM, even in patients still receiving adjuvant therapy [71]. Non-hormonal therapies, including vaginal moisturizers and lubricants, should remain the preferred first-line treatment in breast cancer survivors, particularly during active endocrine therapy. In women with persistent moderate to severe symptoms, low-dose vaginal estrogen, vaginal DHEA, or ospemifene may be considered after multidisciplinary discussion involving the oncologist. In BCS, treatment decisions should always be individualized through shared decision-making involving the patient, gynecologist, and treating oncologist, taking into account estrogen receptor status, current endocrine therapy, recurrence risk, symptom severity, and patient preferences. Low-dose vaginal estrogen, vaginal DHEA, or ospemifene should therefore be considered only in carefully selected patients after multidisciplinary evaluation.


**Key points**—Recommendation for GSM in women with a history of breast cancer.BCS with GSM: Nonhormonal treatments—such as vaginal moisturizers and lubricants—should be first-line therapy, especially during or after endocrine treatments. For moderate to severe symptoms unresponsive to these, low-dose vaginal estrogens and vaginal DHEA may be considered with oncologist consultation, as emerging evidence shows no increased risk of recurrence. Ospemifene may be a valid option for women who have completed treatment, showing efficacy comparable to vaginal estrogens. Energy-based therapies and vaginal oxygen with hyaluronic acid may be considered in select cases, though more robust evidence is needed to confirm long-term safety in this population *(Strength of Recommendation: Moderate)*.


## 9. Management of GSM in Gynecological Cancer Survivors

Women treated for gynecological malignancies frequently experience moderate to severe GSM owing to surgical menopause, chemotherapy-induced ovarian failure, pelvic radiotherapy, or combined multimodal treatments. These patients often present with severe vaginal dryness, dyspareunia, introital stenosis, vaginal shortening, fibrosis, and urinary symptoms, which substantially impair quality of life and sexual function [8]. Management should be individualized according to cancer type, recurrence risk, previous oncological treatment, symptom severity, and patient preferences. Non-hormonal therapies, including vaginal moisturizers and lubricants, should be considered the initial therapeutic approach. Regular sexual activity or vaginal dilator therapy may help reduce radiation-induced vaginal stenosis and maintain vaginal elasticity in women undergoing pelvic radiotherapy. Current evidence suggests that low-dose vaginal estrogen therapy may be considered in women treated for low-risk endometrial cancer after completion of oncological treatment, particularly when non-hormonal therapies fail to provide adequate symptom relief [82]. Available observational studies have not demonstrated an increased risk of recurrence in appropriately selected patients, although long-term randomized evidence remains lacking. Hyaluronic acid and laser therapy have been used in these patients with some benefit for GSM symptoms [83,84]. Similarly, women treated for cervical cancer, who generally have non-estrogen-dependent disease, may also be candidates for local hormonal therapy following multidisciplinary discussion [n]. In contrast, evidence remains more limited for ovarian cancer survivors, particularly in patients with hormone-sensitive tumors. Therefore, treatment decisions should be individualized in collaboration with the treating oncologist. Women who have undergone pelvic radiotherapy require particular attention because radiation-induced fibrosis, vascular damage, and reduced tissue elasticity frequently coexist with estrogen deficiency, often necessitating multimodal treatment combining local hormonal therapy when appropriate, vaginal moisturizers, pelvic floor rehabilitation, and vaginal dilator therapy [1].

## 10. Conclusions

GSM is a common yet often overlooked condition that can greatly affect a woman’s quality of life. Its symptoms can vary widely, which can result in underdiagnosis and inadequate treatment. Therefore, clinicians should take a proactive approach to screen for and diagnose GSM, even if patients do not spontaneously report symptoms. Management of GSM should be tailored to each individual, incorporating evidence-based practices along with the woman’s preferences, needs, and access to treatment options (Figure 1). It is crucial to inform women that GSM is a long-term, progressive condition that will not resolve on its own and may recur if treatment is discontinued. Consequently, ongoing management is typically necessary. Additionally, early intervention should be considered even for asymptomatic women who exhibit objective signs of GSM, as this may help delay the onset of symptoms and enhance overall outcomes. A patient-centered, anticipatory approach is vital for ensuring timely, effective, and enduring care for all women impacted by GSM.


**Key points**—Conclusions.Early diagnosis and individualized treatment are essential for optimal GSM management. Because GSM is a chronic condition, maintenance treatment and regular follow-up are recommended to monitor symptoms, vaginal trophism, and treatment adherence.


## Figures and Tables

**Figure 1:**
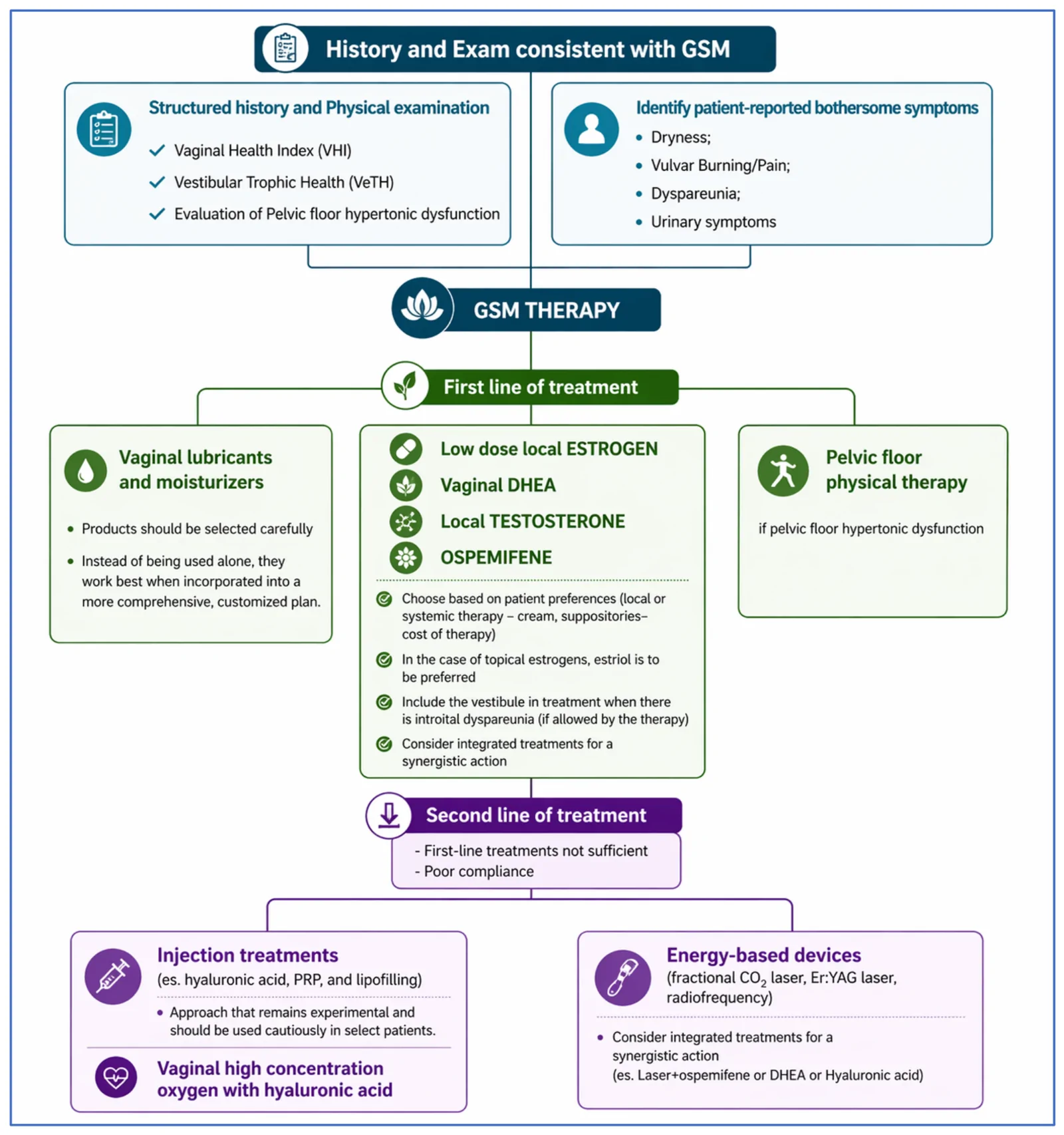
Diagnostic assessment and therapeutic management of genitourinary syndrome of menopause (GSM), including first-line and second-line treatment options.

**Table 1:** Recommendation and evidence.

Strength of Recommendation	Quality of Evidence	Benefits Clearly Outweigh Risks
Strong	High	Benefits clearly outweigh risks
Moderate	Moderate	Most patients would benefit
Weak	Low	Evidence limited or inconsistent
Expert opinion	Very low	Based mainly on clinical expertise

Modified from the Oxford Centre for Evidence-Based Medicine (CEBM); https://www.cebm.ox.ac.uk/resources/levels-of-evidence/ocebm-levels-of-evidence (accessed on 20 April 2026).

**Table 2:** GSM symptoms clusters.

Genital/Vaginal, Vulvar Cluster	Sexual Cluster	Urinary Cluster
Dryness Burning Irritation Itching Soreness/tenderness Vaginal discharge	Dyspareunia Reduced lubrification/ vaginal dryness Bleeding after intercourse Sexual dysfunction	Dysuria Urinary urgency Recurrent urinary tract infections Urinary Incontinence

**Table 3:** Standard administration regimens for vaginal topical hormonal treatments available on the Italian market for the management of GSM.

	Types of Preparations	Starting Dose *	Maintenance Dose *
Estradiol-based formulations Estriol-based formulations Promestriene-based formulations Estriol + *Lactobacillus acidophilus* KS400	Estradiol vaginal tablets Estradiol vaginal ring Estriol vaginal tablets Estriol vaginal tablets Estriol vaginal gel Estriol vaginal cream Promestriene vaginal tablets Estriol + *Lactobacillus acidophilus* vaginal tablet	1 tab/10 mcg/day every day for 3 weeks 1 ring release 7.5 mcg/day (change the ring every three months) 1 tab/30 mcg/day every day for 2–3 weeks 1 tab/100 mcg/day every day for 3 weeks 1 g/50 mcg/day every day for 3 weeks 1 g/125 mcg/day every day for 3 weeks 1 tab/10 mcg/day every day for 20 days 1 tab/30 mcg + ≥10^7^ UFC/day every day for 20 days	1 tab/10 mg/day twice week 1 tab/30 mg/day twice week 1 tab/100 mg/day twice week 1 g/50 mcg/day twice week 1 g/125 mcg/day twice week Up to medical consult Up to medical consult

* The initial dose may require extension beyond 2–3 weeks, with some women needing daily treatment for a longer duration to significantly reduce symptoms before moving to biweekly or triweekly maintenance treatments.

## Data Availability

No new data were created or analyzed in this study. Data sharing is not applicable to this article.

## Abbreviations

The following abbreviations are used in this manuscript:

GSM	Genitourinary syndrome of menopause
DHEA	Dehydroepiandrosterone
VHI	Vaginal health index
VeTH	Vestibular trophic health
SUI	Stress urinary incontinence
SERM	Selective estrogen receptor modulator
AEs	Adverse effects
RF	Radiofrequency
CO_2_	Carbon dioxide
Er:YAG	Erbium-doped yttrium aluminum garnet
FSFI	Female sexual function index
RCTs	Randomized controlled trials
EBD	Energy based devices
HA	Hyaluronic acid
PRP	Platelet-rich plasma
MFAT	Micro-fragmented adipose tissue
BCS	Breast cancer survivors
AIs	Aromatase inhibitor
